# Pyroptosis at the forefront of anticancer immunity

**DOI:** 10.1186/s13046-021-02065-8

**Published:** 2021-08-24

**Authors:** Reid Loveless, Ryan Bloomquist, Yong Teng

**Affiliations:** 1 Department of Oral Biology and Diagnostic Sciences, Dental College of Georgia, Augusta University, Augusta, GA 30912 USA; 2 Department of Restorative Sciences, Dental College of Georgia, Augusta University, Augusta, GA 30912 USA; 3 Department of Hematology and Medical Oncology, Winship Cancer Institute, Emory University, 201 Dowman Dr, Atlanta, GA 30322 USA

**Keywords:** Pyroptosis, Antitumor immunity, Gasdermin, cancer, The immune landscape

## Abstract

Tumor resistance to apoptosis and the immunosuppressive tumor microenvironment are two major contributors to poor therapeutic responses during cancer intervention. Pyroptosis, a lytic and inflammatory programmed cell death pathway distinct from apoptosis, has subsequently sparked notable interest among cancer researchers for its potential to be clinically harnessed and to address these problems. Recent evidence indicates that pyroptosis induction in tumor cells leads to a robust inflammatory response and marked tumor regression. Underlying its antitumor effect, pyroptosis is mediated by pore-forming gasdermin proteins that facilitate immune cell activation and infiltration through their release of pro-inflammatory cytokines and immunogenic material following cell rupture. Considering its inflammatory nature, however, aberrant pyroptosis may also be implicated in the formation of a tumor supportive microenvironment, as evidenced by the upregulation of gasdermin proteins in certain cancers. In this review, the molecular pathways leading to pyroptosis are introduced, followed by an overview of the seemingly entangled links between pyroptosis and cancer. We describe what is known regarding the impact of pyroptosis on anticancer immunity and give insight into the potential of harnessing pyroptosis as a tool and applying it to novel or existing anticancer strategies.

## Background

While long evading discovery, the existence and physiological significance of programmed cell death (PCD) pathways distinct from apoptosis have garnered increasing interest in recent years, in part, due to the high prevalence of apoptosis resistance in tumors [1]. Of these different forms, pyroptosis, a necrotic and lytic PCD, has distinguished itself from others by its ability to induce a powerful inflammatory response [2]. Similar to necroptosis, a programmed form of necrosis, pyroptosis is believed to exist principally as a defense against pathogens by triggering an antimicrobial response through the release of immunogenic cellular content, including damage-associated molecular patterns (DAMPs) and inflammatory cytokines [3]. Unlike necroptosis, which is mediated by mixed lineage kinase domain-like pseudokinase (MLKL) and caspase-independent [4], pyroptosis is mediated by gasdermin (GSDM) family proteins and, like apoptosis, largely caspase-dependent [5]. Other forms of regulated necrosis, such as ferroptosis, have also recently emerged [6–10] and are compared alongside necrosis and apoptosis in Table 1.

**Table 1:** Comparison of select cell death forms

	Inducers	Key constituents	Characteristics	Cell release	Immune features
Apoptosis (PCD)	TNF-α, FasL, TRAIL, Hypoxia, Irradiation, Heat shock	Bcl-2 protein family, P53, Caspase-2/3/6/7/8/9/10, HSPs	Plasma membrane blebbing, Reduced cellular volume, Nuclear fragmentation and chromatin condensation	In certain cases: DAMPs (e.g., dsDNA, HMGB1, ATP, calreticulin)	Mostly anti-inflammatory. Pro-inflammatory in cases involving the release of DAMPs
Pyroptosis (PCD)	DAMPs, PAMPs, Microbial infection	GSDM protein family, Caspase-1/3/4/5/8/11, Inflammasomes	Plasma membrane rupture and pore formation, Cytoplasmic swelling, Chromatin condensation	Intracellular content, DAMPs (e.g., IL-18, IL-1β, dsDNA, ATP, HMGB1)	Pro-inflammatory
Necroptosis (PCD)	TNF-α, TRAIL, Fas ligand, Microbial infection	MLKL, RIPK1/3 (Necrosome), TRADD	Plasma membrane rupture, Cytoplasmic and organelle swelling, Moderate chromatin condensation	Intracellular content, DAMPs (e.g., IL-1α, IL-33, IL-6, HSPs)	Mostly pro-inflammatory. Anti-inflammatory in certain cases
Ferroptosis (PCD)	ROS from iron accumulation and lipid peroxidation	GPX4, System X_C_^−^, GSH, ACSL4	No plasma membrane blebbing or rupture, Small mitochondria with ruptured outer membrane, Normal nucleus	DAMPs (e.g., HMGB1, dsDNA), lipid oxidization products (e.g., 4-HNE, LTB4)	Pro-inflammatory
Necrosis (Accidental)	Microbial infection, Toxins, Trauma, Ischemia, Thermal stress	Unspecific	Plasma membrane rupture, Cytoplasmic and organelle swelling, Random DNA degradation	Intracellular content, DAMPs (e.g., IL-1α, IL-33, dsDNA, ATP, HMGB1)	Pro-inflammatory

ACSL4, Acyl-CoA synthetase long-chain family member 4; Bcl-2, B-cell lymphoma 2; DAMPs, danger-associated molecular patterns; dsDNA, double-stranded DNA; GPX4, glutathione peroxidase 4; GSDM, gasdermin; GSH, glutathione; HMGB1, high-mobility group box protein 1; HSPs, heat shock proteins; IL, interleukin; LTB4, Leukotriene B4; MLKL, mixed lineage kinase domain-like protein; PAMPs, pathogen-associated molecular patterns; PCD, programmed cell death; RIPK1/3, receptor-interacting serine/threonine-protein kinase 1/3; ROS, reactive oxygen species; TNF-α, tumor necrosis factor-alpha; TRADD, TNFR-associated death protein; TRAIL, TNF-related apoptosis-inducing ligand; X_C_^−^, cysteine/glutamate transporter receptor; 4-HNE, 4-Hydroxynonenal

The quest to overcome cancer and its grave global consequences has repeatedly led us to face the cheat of death and detection by cancer cells. While still a relatively obscured process, pyroptosis represents a potentially harnessable and potent means to not only bypass apoptosis resistance but to activate tumor-specific immunity and/or enhance the effectiveness of existing therapies. Here, we discuss the current knowledge of pyroptosis in the context of anticancer immunity to give insight into its potential to fight cancer.

## Pyroptosis at a glance

Pyroptosis was first described in the 1990s in macrophages infected with *S. enterica* serovar Typhimurium (*S. Typhimurium*) [11] and *S. flexneri* [12]. Although originally thought to be a process of apoptosis, further study revealed that this bacteria-induced cell death was heavily dependent on caspase-1 [13], a caspase that is not involved in apoptosis execution (i.e., caspase-3). Shortly afterward in 2001, this PCD was coined pyroptosis, or “fiery falling”, to describe the release of pro-inflammatory signals by the dying cells. Pyroptotic cells share several features with apoptotic cells, such as chromatin condensation and DNA fragmentation, but are distinguishable by their intact nucleus, pore formation, cell swelling, and osmotic lysis (Table 1) [14]. Generally, pyroptotic cell rupture is achieved through the caspase-mediated activation of pore-forming GSDM proteins following the binding of DAMPs or pathogen-associated molecular patterns (PAMPs) [15]. These same caspases may also directly or indirectly contribute to the maturation of pro-inflammatory cytokines that, alongside DAMPs, initiate or perpetuate an inflammatory response when released.

Although serving an important protective role in pathogen resolution, pyroptosis has been implicated as a complicating factor in several human diseases, such as cardiovascular disease [16], neurodegenerative disease [17], and HIV/AIDS [18]. Metabolic disorders like diabetes may also be promoted by pyroptosis through chronic inflammation and the production of insulin-interfering cytokines [19]. In cancer, the role of pyroptosis appears to be double-edged. On one side, pyroptosis can rapidly lead to tumor regression and, on the other, it can facilitate the development of the tumor microenvironment. Hence, cancer cells may either suppress or incite pyroptosis to support their progression depending on the context.

## Molecular mechanisms of pyroptosis

**Fig. 1:**
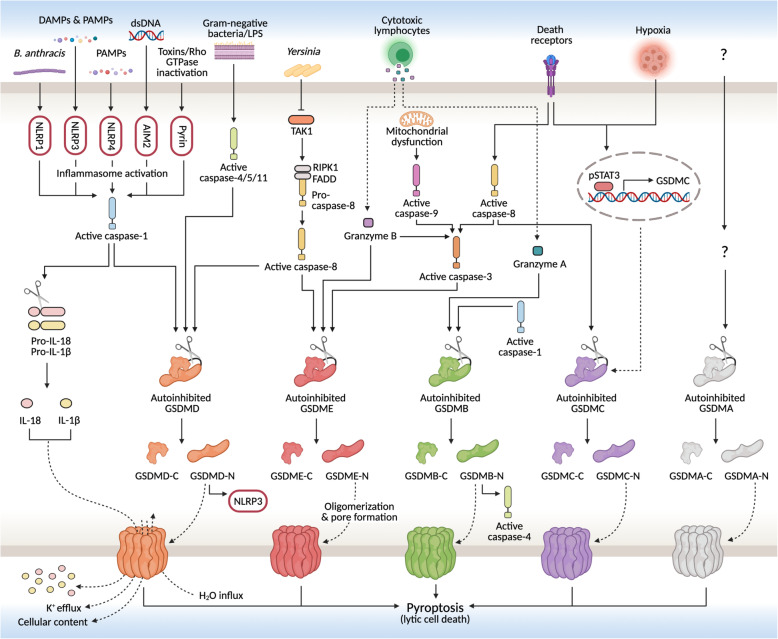
Schematic of pyroptosis signaling pathways. The canonical inflammasome pathway to pyroptosis is induced by various stimuli and results in caspase-1 activation, while the non-canonical pathway is induced by LPS and results in caspase-4/5 activation. Both activated caspase-1 and caspase-4/5 cleave autoinhibited GSDMD at its linker region to free the N-terminal domain of GSDMD (GSDMD-N) from its repressor C-terminal domain (GSDMD-C). GSDMD-N then translocates to the plasma membrane and undergoes oligomerization and pore formation, which causes an increase in osmotic pressure and eventually cell lysis. Pore formation also facilitates the release of intracellular content and the inflammatory cytokines IL-18 and IL-1β following their activation by caspase-1. Through alternative pathways, GSDMD may also be cleaved by caspase-8, similar to GSDME, which can additionally be cleaved by caspase-3 and granzyme B. Aside, GSDMD-N and GSDMB-N can also respectively activate NLRP3 or caspase-4. In the other alternative pathways, GSDMB is cleaved by caspase-1 or granzyme A, while GSDMC is cleaved by caspase-8 and transcriptionally upregulated under hypoxia through pSTAT3 interaction with programmed death-ligand 1. The mechanisms of GSDMA-mediated pyroptosis have yet to be elucidated. AIM2, absent in melanoma 2; DAMPs, danger-associated molecular patterns; FADD, Fas-associated death domain protein; GSDMA/B/C/D/E, gasdermin A/B/C/D/E; IL, interleukin; LPS, lipopolysaccharides; NLRP1/3/4, NLR family pyrin domain-containing 1/3/4; PAMPs, pathogen-associated molecular patterns; RIPK1, receptor-interacting serine/threonine-protein kinase 1; pSTAT3, phospho-signal transducer and activator of transcription 3; TAK1 (also known MAP 3 K7), transforming growth factor beta-activated kinase 1

Although the number of known pyroptosis pathways is likely to increase in the future, there are currently two principal and several alternative pathways that have been elucidated to date (Fig. 1). In the principal pathways, pyroptosis is induced by GSDMD and involves inflammatory caspase-1 (canonical pathway) or caspase-4/5 (or mouse caspase-11) (non-canonical pathway). Of the alternative pathways, the most widely regarded is GSDME-induced pyroptosis through caspase-3 [5], though different pathways involving other GSDM family members and caspases or granzymes have also been reported. Structurally, GSDMA, GSDMB, GSDMC, GSDMD, and GSDME are all comprised of an N-terminal pore-forming domain and a C-terminal regulatory domain that are joined by a linker region [20]. Under normal conditions, the linker region allows the C-terminal domain to fold over the top of the N-terminal domain and functionally inhibit its lethal activity. Cleavage at the linker site by caspases or granzymes, however, relinquishes this auto-inhibitory structure and leads to the translocation of the N-terminal domain fragment into the plasma and mitochondrial membranes. Once bound, the N-terminal domain oligomerizes and forms β-barrel transmembrane pores that facilitate the secretion of pro-inflammatory content, like interleukin (IL)-1β and IL-18, and cause cell lysis through osmotic barrier disruption [21]. In the subsequent sections, a summary of the steps involved in each of the pathways leading to pyroptosis is provided.

### Canonical inflammasome pathway

In the canonical inflammasome pathway to pyroptosis, recognition of DAMPs (e.g., fibrinogen, heat shock proteins, DNA) and/or PAMPs (e.g., flagellin, glycans, lipopolysaccharides (LPSs)) by pattern recognition receptors (PRRs) leads to the activation of respective cytosolic signaling complexes called inflammasomes, which are typically comprised of a sensor protein, adaptor, and effector caspase [22]. Although a variety of PRRs, like NOD-like receptors (NLRs) and toll-like receptors (TLRs), are involved in this process, only a subset of these is known to be able to directly assemble inflammasomes and activate the cysteine protease caspase-1 [23]. Specifically, the PRRs/inflammasome sensors in this subset include NLR family pyrin domain-containing (NLRP)1, NLRP3, NLRP4, absent in melanoma 2 (AIM2), and Pyrin. Following their activation, the majority of these sensors interact with the adaptor protein apoptosis-associated speck-like protein containing CARD (ASC), which activates caspase-1 through pro-caspase-1 recruitment and cleavage. In addition to freeing and activating the lethal N-terminal domain of GSDMD (GSDMD-N), caspase-1 also matures pro-IL-1β and pro-IL-18 into IL-1β and IL-18, which are released through the necrotic membrane pores formed by GSDMD-N [24].

### Non-canonical inflammasome pathway

In contrast to the canonical inflammasome pathway, the non-canonical inflammasome pathway is independent of caspase-1 and instead reliant on caspase-4 and -5 in humans and caspase-11 in mice [25]. The activation of these caspases occurs through the direct binding of LPS to respective pro-caspases and bypasses the need for inflammasome sensors. Originating from gram-negative bacteria, cytoplasmic delivery of LPS may occur through infection or membrane vesicles. Although these caspases do not activate IL-1β and IL-18 directly, their triggering of pyroptosis through GSDMD cleavage leads to an efflux of potassium ions that activates the NLRP3 inflammasome and upregulates the action of caspase-1 [26].

### Alternative pathways

It was revealed that in certain contexts, such as chemotherapy or targeted cancer therapy, a pathway from apoptosis to pyroptosis can be induced through caspase-3 [5]. Although principally associated with apoptosis execution and morphological changes, caspases-3 can mediate pyroptosis through the cleavage of GSDME, which similarly leads to GSDME-N pore formation and membrane permeabilization. When GSDME levels are high, pyroptosis is rapidly prompted following caspase-3 activation, but when GSDME levels are low, apoptosis is prompted instead [5]. Considering that most of the proteases involved in pyroptosis can also mediate apoptosis when their respective GSDM protein is absent [27, 28], it is suggestive that the balance between pyroptosis and apoptosis is largely dependent on GSDM protein levels. This notion requires further evidence, however, as it is contradicted by studies challenging the role of GSDME in pyroptosis [29, 30]. Several other alternative pyroptosis pathways have also been reported and, in brief, include GSDMD cleavage by caspase-8 [31], GSDME cleavage by caspase-8 [32] or granzyme B (GzmB) [33], GSDMB cleavage by caspase-1 [34] or granzyme A (GzmA) [35], GSDMC cleavage by caspase-8 and transcriptional upregulation by hypoxia-activated programmed death-ligand 1 (PD-L1) and pSTAT3 [36], and GSDMA pore formation through an unknown mechanism [37].

## Pyroptosis and its constituents in cancer

The obscure role of pyroptosis in cancer appears to be contextual and dependent on cell type, genetics, and duration of pyroptosis induction. Following aberrant expression and prolonged activity, GSDMs, inflammasomes, and/or pro-inflammatory cytokines can contribute to tumor pathology by inducing immunosuppressive cells, promoting epithelial-to-mesenchymal transition, and/or upregulating matrix metalloproteinases for extracellular matrix remodeling [38]. Recently, it has been found that pyroptosis can fuel tumor progression in colorectal cancer (CRC) by increasing the expression of proliferating cell nuclear antigen through high-mobility group box protein 1 (HMGB1) release [39]. In the hypoxic regions of MDA-MB-231 xenografts in nude mice, a PD-L1 mediated apoptosis to pyroptosis switch has also been reported to facilitate chronic tumor necrosis [36], which can promote tumor growth and impede antitumor immunity [40]. Juxtaposing these effects, however, pyroptosis can also commence tumor suppression and execution [5, 33, 41–43]. In hepatocellular carcinoma (HCC) cells, for example, pyroptosis induction through NLRP3 inflammasome activation significantly impeded metastatic potential in vitro and tumor growth in vivo in a mouse xenograft model [44]. The idea that pyroptosis suppression confers a selective advantage in HCC cells is further supported by the observation that caspase-1 mRNA and protein levels are actively downregulated in human HCC tissues and cell lines [45].

Given the dual role of pyroptosis, its molecular components are, as one would expect, abnormally and differentially expressed across different cancers (Table 2). GSDMs, for instance, are deregulated in breast, gastric, cervical, and lung cancers, among others, and have been shown to control proliferation, metastasis, therapeutic resistance, and antitumor immunity while acting as either oncogenes or tumor suppressors [65, 66]. In gastric cancer (GC), *GSDMD* expression was markedly decreased and resulted in enhanced tumor proliferation both in vitro and in vivo, possibly by accelerating S/G2 cell transition [57]. Conversely, GSDMD protein levels were remarkably increased in non-small cell lung cancer (NSCLC) compared to adjacent controls and were associated with greater tumor size, more advanced tumor node metastasis stages, and, in lung adenocarcinoma (LUAD), poorer prognosis [27]. Moreover, GSDMD knockdown in NSCLC cells attenuated their proliferation through apoptosis induction and EGFR/Akt signaling inhibition. Similar to *GSDMD*, *GSDME* expression was also decreased in GC, as well as in breast cancer and CRC [47, 59, 67]. In CRC particularly, *GSDME* knockdown increased cellular invasiveness and colony numbers, whereas GSDME overexpression decreased cell growth and colony formation [51]. When examining the surgical specimens of primary GC, *GSDMC* expression was seen only in certain cases, though contrastingly upregulated in CRC, where it promoted carcinogenesis and proliferation in vitro and tumor growth in vivo [50]. Higher levels of GSDMB have also been correlated with higher rates of metastasis and lower survival rates in breast cancer patients [46]. Among other pyroptosis constituents, AIM2 expression was markedly decreased or absent in the majority of CRC tumors observed and tied to poor patient outcomes [52]. Low AIM2 levels also correlated with more advanced tumor progression in HCC, whereas AIM2 overexpression attenuated cell proliferation and invasion [61]. NLRP1 levels were similarly diminished in CRC tumoral tissues and linked to increased metastasis and poor survival [54]. Nevertheless, NLRP1 has also been implicated in tumor support. In melanoma, for example, NLRP1 was found to contribute to acquired drug resistance [62], and in breast cancer, was overexpressed in primary tissues and associated with lymph node metastasis [49]. In mice, NLRP1 also promoted breast cancer proliferation, invasion, metastasis, and tumorigenicity [49]. Moving on, caspase-1 mRNA levels were significantly decreased in the breast cancer tissues of patients [48], and loss of caspase-1 was associated with prostate [64] and CRC [53] tumorigenesis. Despite its apparent tumor-suppressing role in these cancers, caspase-1 expression was markedly increased in human glioma tissues and suggested to play a key role in glioma cell proliferation and migration through its control of pyroptosis and subsequent contribution to the local tumor microenvironment [60].

**Table 2:** Expression of select pyroptotic components in cancers and their associated consequence(s)

Cancer type	Setting	Cancer line/ model/tissue	Pyroptotic component	Relative expression	Associated consequence(s) of relative expression	Ref
Breast cancer	In vivo: human	Primary tissue	GSDMB	Increased	↑ Metastasis & ↓ Patient survival	[46]
	In vivo: human	Primary tissue	GSDME	Decreased	↑ Metastasis	[47]
	In vitro: -	MDA-MB-231		Decreased*	↑ Invasion	[47]
	In vivo: human	Primary tissue	Caspase-1	Decreased	N/A	[48]
	In vitro: -	MDA-MB-231		Decreased*	↑ Proliferation & Invasion	[48]
	In vivo: human	Primary tissue	NLRP1	Increased	↑ Metastasis	[49]
	In vivo: mouse	MCF-7		Increased	↑ Tumorigenicity & Invasion	[49]
Colorectal cancer	In vivo: mouse	LoVo	GSDMC	Increased	↑ Tumor growth	[50]
	In vitro: -	DLD-1, LoVo		Increased	↑ Proliferation	[50]
	In vivo: human	Primary tissue	GSDME	Decreased	N/A	[51]
	In vitro: -	HCT116		Decreased*	↑ Cell growth	[51]
	In vivo: human	Primary tissue	AIM2	Decreased	↓ Patient survival	[52]
	In vivo: mouse	AOM-DSS	Caspase-1	Decreased*	↑ Tumorigenesis	[53]
	In vivo: human	Primary tissue	NLRP1	Decreased	↑ Metastasis & ↓ Patient survival	[54]
Gastric cancer	In vitro: -	MKN28	GSDMA	Decreased	↑ Cell growth	[55]
	In vivo: human	Primary tissue	GSDMB	Decreased	N/A	[35]
	In vitro: -	MKN28		Increased*	No change in cell growth	[56]
	In vitro: -	MKN28	GSDMC	Increased*	↓ Cell growth	[56]
	In vivo: human	Primary tissue	GSDMD	Decreased	N/A	[57]
	In vivo: mouse	BGC823		Increased*	↓ Tumor growth	[57]
	In vivo: human	Primary tissue	GSDME	Decreased	N/A	[58]
	In vivo: mouse	AOM		Decreased*	No changes reported	[59]
Glioma	In vivo: human	Primary tissue	Caspase-1	Increased	N/A	[60]
	In vitro: -	U87, T98G		Increased*	↑ Proliferation & Mobility	[60]
Hepatocellular carcinoma	In vivo: human	Primary tissue	AIM2	Decreased	↑ Tumor progression	[61]
	In vitro: -	HuH-7		Increased*	↓ Proliferation & Invasion	[61]
Lung cancer	In vivo: human	Primary tissue	GSDMD	Increased	↑ Tumor size &	[27]
	In vivo: mouse	PC9		Decreased*	↑ Metastasis stage ↓ Tumor growth	[27]
Melanoma	In vitro: -	1205Lu	NLRP1	Increased*	↑ TMZ resistance	[62]
	In vivo: human	Primary tissue		Decreased	N/A	[63]
	In vivo: mouse	1205Lu		Decreased*	↓ Tumor growth	[63]
Prostate cancer	In vivo: human	Primary tissue	Caspase-1	Decreased	N/A	[64]

* indicates expression was forced or a consequence of experimental treatment during functional studies. The term relative expression is used broadly here and includes mRNA and/or protein level expression depending on the study. AIM2, absent in melanoma 2; AOM, azoxymethane; DSS, dextran sodium sulfate; GSDMA/B/C/D/E, gasdermin A/B/C/D/E; NLRP1, NLR family pyrin domain-containing 1; TMZ, temozolomide

Needless to say, elucidating the relationship between pyroptosis and cancer will continue to require extensive investigation. Considering the lack of consensus across studies, one notable challenge will be to discern and piece together the tumor-specific roles and regulation of each pyroptotic molecular component. With multiple pathways leading to pyroptosis and multiple constituents overlapping, it is suggestive that characterizing each pathway’s overall tumor-specific effect, rather than the individual effects of each component, may be a more effective strategy to understand and/or anticipate a tumor’s modulation of pyroptosis. Nevertheless, as new pyroptosis pathways are still being discovered, gaps in our knowledge may prevent us from grasping any larger modulatory themes until all respective signaling pathways are elucidated and accordingly organized within the current schema or a new one.

## Ties between pyroptosis and anticancer immunity

The ability of a cell’s death to elicit an adaptive immune response is known as immunogenic cell death (ICD). Particularly, the immunogenic potential of a dying cancer cell is defined by its antigenic and adjuvant features, such as the presence of tumor-associated antigens and the release of endogenous DAMPs, respectively [68, 69]. Unlike apoptosis, which is fundamentally an immune tolerant process, pyroptosis possesses the molecular machinery to elicit a robust inflammatory response and is suggested to be a form of ICD in some cases [33]. While the link between pyroptosis and anticancer immunity is not yet clear, a growing number of studies demonstrate that pyroptosis-mediated tumor clearance is achieved through amplifying immune activation and function. Moreover, in addition to being triggered spontaneously through different stressors and apoptosis-to-pyroptosis switches, tumor cell pyroptosis can be directly induced by certain immune cells, suggesting that pyroptosis may participate in a positive feedback loop in antitumor immunity. In the following sections, the most recent investigations implicating pyroptosis in anticancer immunity are highlighted according to the GSDM protein involved.

### GSDMA

Using the cancer-imaging probe phenylalanine trifluoroborate (Phe-BF3) in combination with gold nanoparticle (NP) delivery, Wang et al. reported successfully delivering a mouse isoform of GSDMA, Gsdma3, selectively into human HeLa (cervical), mouse EMT6 (mammary), and mouse 4 T1 (mammary) cancer cells, leading to pyroptosis in 20–40% of the cells depending on the cell line [70]. When this delivery system was applied to BALB/c mice subcutaneously implanted with 4 T1 or EMT6 cells after two weeks of growth, three rounds of treatment with NP–Gsdma3 and Phe-BF3, either by intravenous or intratumoral injection, resulted in marked tumor shrinkage; and after 25 days, tumor burden was negligible. In comparison, no tumor shrinkage was observed when NP–Gsdma3 or Phe-BF3 were injected alone, or when a mutant non-pore-forming NP–Gsdma3 and Phe-BF3 were injected together, suggesting Gsdma3 function to be necessary to the observed antitumor effect. Interestingly, in NP–Gsdma3 and Phe-BF3 treated BALB/c mice, it was found that pyroptosis in less than 15% of 4 T1 tumor cells was sufficient to eliminate the entire mammary tumor graft. This tumor regression effect was absent in *Nu/Nu* mice lacking mature T cells, however, strongly indicating that the tumor elimination effect of Gsdma3-mediated pyroptosis was, at least in part, dependent on the immune system. Accordingly, an increase in CD3^+^ T cell infiltration, as well as a decrease in CD4^+^FOXP3^+^ T regulatory cells in BALB/c mice, were seen only in the 4 T1 tumors treated with NP–Gsdma3 and Phe-BF3. Furthermore, depletion of CD4^+^ and CD8^+^ cell populations in this treatment model prevented tumor regression, implying that both CTLs and CD4^+^ T helper cells play an indispensable role during pyroptosis-induced tumor clearance. When compared with PBS control 4 T1 tumors, further analysis also revealed that while CD4^+^, CD8^+^, natural killer (NK), and M1 macrophage cell populations increased in NP–Gsdma3 and Phe-BF3 treated tumors, the populations of monocytes, neutrophils, myeloid-derived suppressor cells, and M2 macrophages decreased. In addition to increased IL-1β, IL-18, and HMGB1 serum and tumor levels, numerous immunostimulatory and antitumor effector genes (e.g., *Cd69*, *Gzma*, *Gzmb*) were found to be upregulated and various immunosuppressive and protumor genes (e.g., *Csf1*, *Vegfa*, *Cd274*) downregulated in the 4 T1 tumors treated with NP–Gsdma3 and Phe-BF3 in BALB/c mice [70].

### GSDMD

Focusing their attention on cytotoxic T lymphocytes (CTLs), Xi and colleagues examined CTLs’ expression of GSDM genes in relation to CD8^+^ T cell markers in LUAD, lung squamous cell carcinoma (LUSC), and melanoma tumor samples using data from The Cancer Genome Atlas (TCGA) [71]. Of the five GSDM gene members, only *GSDMD* expression showed a positive correlation with CD8^+^ T cell marker genes (e.g., *CD8A*, *CD8B*, *PRF1*, *GZMA*, *GZMB*, and *IFNG*) in CTLs across all three tumor cohorts. A positive correlation between *GSDMD* and *CD8A*, *GZMB*, and *IFNG* expression in CTLs was also seen in many other tumor types and in 30 primary tumor samples from patients with NSCLC, further confirming the associations seen from TCGA. Further study revealed that the expression of *GSDMD* in activated CTLs from OT-1 mice was significantly increased compared with naïve T lymphocytes. Similarly, human CD8^+^ T cells upregulated *GSDMD* following their activation, and in LUAD and LUSC tissue samples, high levels of GSDMD protein were seen in tumor-infiltrating lymphocytes (TILs). In both OT-1 and human activated CD8^+^ T cells, the activation of caspase-11 or caspase-4 were respectively enhanced and targeting them with short hairpin RNA attenuated GSDMD cleavage. When activated OT-1 T cells were co-cultured with ovalbumin-expressing Lewis lung carcinoma (3LL-OVA) cells, the co-localization of GSDMD and GzmB was observed in the CTLs near their immune synapses; moreover, CTL cytotoxicity towards 3LL-OVA cells was diminished following *GSDMD* knockdown. Similar results were recorded using human CTLs and an H1299 NSCLC cell line [71]. Considering that one critical way by which CTLs kill tumor cells is through the release of cytotoxic molecules into the immune synapse that they form, it was speculated that the delivery of GSDMD and GzmB into effector cancer cells may have been the mechanism underlying CTL cytotoxicity seen in this study [71].

### GSDMB

Shortly after Xi and colleagues’ report, a mechanism of NK- and CTL-induced tumor cell pyroptosis through granzyme release was reinforced by several studies [33, 35, 72]. In contrast to Xi et al., however, Zhou et al., for example, implicated the involvement of GzmA and GSDMB, rather than GzmB and GSDMD, in the cell lines they examined, supporting the notion that a cell’s response to granzymes and GSDMs is contextual and dependent on the cell’s type [35, 71]. Specifically, it was found that forced expression of GSDMB but no other GSDM members in human embryonic kidney (HEK)-293 T cells lacking endogenous expression of GSDMs conferred pyroptotic killing of 293 T cells by co-cultured human NK-92MI cells [35]. Interestingly, GSDMB-mediated killing by NK cells appeared to be caspase-independent, as treatment with a pan-caspase inhibitor had no effect. The inhibition of granzymes or NK cell degranulation and perforin, however, not only blocked NK cell-induced pyroptosis but also GSDMB cleavage in 293 T cells. Of the five human granzymes in HEK-293F cells, it was found that only GzmA rapidly cleaved GSDMB in a pattern similar to that seen in NK cell-killing assays. When GzmA was electroporated into GSDMB-reconstituted 293 T cells, extensive GSDMB cleavage and pyroptotic killing resulted; but when a protease-deficient GzmA S212A mutant was electroporated or a non-cleavable GSDMB K244A mutant or K229A/K244A double mutant was expressed, pyroptosis induction was significantly diminished. Similarly, GzmA-mediated cleavage of GSDMB was required under physiological conditions for NK cell pyroptotic killing of 293 T cells, and any disruptions to the cleavage, such as GSDMB mutant expression, pointed 293 T cells towards pyroptosis resistance. In human cancer cell lines endogenously expressing GSDMB, specifically OE19 (esophageal carcinoma), SW837 (CRC), and SKCO1 (CRC), it was further shown that GzmA delivery through electroporation or perforin was sufficient to induce GSDMB-mediated pyroptosis [35].

Notably, other cancer cell lines with inappreciable GSDMB levels, such as OE33 (esophageal carcinoma cells) and HCC1954 (breast cancer cells), could be transcriptionally induced to increase GSDMB expression through exposure to cytokines typically released by activated cytotoxic lymphocytes, like interferon-gamma (IFN-γ) and tumor necrosis factor-alpha (TNF-α) [35]. In turn, IFN-γ priming significantly enhanced pyroptotic cell death across a number of these cell lines, though this effect was ultimately dependent on GzmA. Similar to their incubation with NK-92MI cells, 293 T cells expressing CD19 and GSDMB were found to undergo GSDMB cleavage and pyroptosis in response to incubation with human anti-CD19 chimeric antigen receptor (CAR) -T cells. This cleavage and pyroptosis induction, however, did not occur when a non-cleavable version of GSDMB was expressed in 293 T cells or when *GZMA* was knocked down in the CAR-T cells. Moving forward, the group demonstrated that, although *GSDMB* possesses no orthologs in mice, CTLs generated from OT-1 transgenic mice can use mouse GzmA (mGzmA) to cleave human GSDMB and induce pyroptosis in mouse MC38 CRC cells expressing human GSDMB. Applying this knowledge to an in vivo model, the group found no appreciable differences in the growths of engrafted mouse CT26 CRC cells in BALB/c mice whether human GSDMB was reconstituted in the cells or not, however. It was subsequently put forth that the recognition of CT26 tumor cells by CTLs in the model may have been impeded by programmed cell death protein 1 (PD-1)–programmed-death ligand 1 (PD-L1) interaction, thus, preventing CTL delivery of mGzmA into target CT26 cells and induction of CT26 cell pyroptosis. Remarkably, by blocking PD-1-PD-L1 binding in the model through PD-1 antibody injection, the group was able to slightly reduce the growth of control CT26 tumors and almost entirely suppress the growth of human GSDMB expressing CT26 tumors. Partial inhibition of tumor growth was also seen in CT26 tumors expressing the GzmA-resistant double mutant form of GSDMB under the PD-1 antibody condition, but only to an extent near that of control tumors. The group also reported similar findings using a more aggressive B16-F10 melanoma tumor model in C57BL/6 mice [35]. Taken together, these findings not only demonstrated that GSDMB-mediated pyroptosis acts downstream of GzmA but that cytotoxic lymphocytes may deliver GzmA into GSDMB-expressing cancer cells to facilitate antitumor immunity.

### GSDME

Zhang et al. also reported this same mechanism of pyroptosis induction by cytotoxic lymphocytes but pointed to GSDME’s and GzmB’s involvement [33]. Leading to these findings, it was demonstrated that ectopically expressing mouse GSDME (mGSDME) in murine 4T1E breast cancer cells engrafted into immunocompetent BALB/c mice significantly inhibited 4T1E tumor growth and led to an increase in the infiltration of NK cells and tumor-associated macrophages (TAMs) [33]. In addition, NK cell and CD8^+^ TIL expression of GzmB and perforin in these tumors increased, as well as CD8^+^ TIL production of IFN-γ and TNF when stimulated by phorbol 12-myristate 13-acetate and ionomycin. Conversely, expression of non-functional or non-cleavable versions of mGSDME in 4T1E cells significantly mitigated these effects, while *mGSDME* knockout in EMT6 tumors had opposite effects. When 4T1E tumor cells expressing enhanced green fluorescent protein (eGFP) were implanted in these mice, the numbers of eGFP-positive CD8^+^ TILs were seen to be markedly higher when the 4T1E cells also overexpressed mGSDME. eGFP-positive TILs in mGSDME overexpressing tumors also had higher perforin expression and cytokine production secondary to GFP staining; and a doubling of eGFP-positive TAMs in these tumors compared to controls strongly indicated greater tumor cell phagocytosis, which may have helped promote antitumor adaptive immunity. To probe the connection between GSDME-mediated tumor suppression and immune response, NSG mice lacking mature lymphocytes and perforin-deficient BALB/c mice were separately employed by the group to reveal that the antitumor effect of GSDME was both lymphocyte- and perforin-dependent and implicated the involvement of NK and CD8^+^ T cells. Through further investigation, it was shown that the human NK cell line YT can activate pyroptosis in GSDME-expressing HeLa cells and speculated from experiments using the human neuroblastoma SH-SY5Y cell line that this induction was achieved through GzmB, which not only cleaves GSDME at the same site as caspase-3 but indirectly activates caspase-3. Vaccine/challenge experiments also strongly indicated that pyroptosis was a form of ICD, which is consistent with the increased infiltration and enhanced immune cell function observed during earlier experiments with mGSDME-overexpressing cells [33].

These findings are in accordance with those by Liu et al., which suggested that CAR-T cells can induce GSDME-mediated tumor cell pyroptosis in B leukemic and solid tumor cells through perforin and GzmB release [72]. GzmB was likewise shown to rapidly cleave GSDMB and activate caspase-3 in Luc-Raji and NALM-6 cells, although its release and potential to induce mouse B16 melanoma cell pyroptosis was suggested to be dependent on CAR-T cell tumor antigen affinity and co-signaling domains or its quantity when released, respectively. Treating human-derived macrophages with the supernatants from co-cultured CD19-CAR-T cell and cancer cells (NALM-6, Raji, or primary B leukemic cells) prompted macrophage activation of caspase-1, cleavage of GSDMD, and release of IL-6 and IL-1β. These observations, however, were not seen if the co-cultured cancer cells were deficient in GSDME or the macrophages in caspase-1, GSDMD, or NLRP3. It was also revealed that ATP and HMGB1 in the co-cultured pyroptotic supernatants were respectively sufficient to promote macrophage IL-1β secretion and IL-6 upregulation. In large, these findings foreshadowed those seen in a leukemia CAR-T cell-induced cytokine release syndrome (CRS) mouse model (using Raji or NALM-6 cells in severe combined immunodeficient beige mice), which indicated that CAR-T cell therapy elicited CRS through GSDME-facilitated pyroptosis. This was notion was further supported when primary B leukemic cells from patients before CD19-CAR T cell treatment were analyzed and showed increased GSDME levels to be associated with more severe CRS [72].

Aside, it is worth mentioning that in a separate study, treatment-induced pyroptosis in melanoma cells via GSDME and caspase-3 accordingly promoted HMGB1 release and was directly tied to the infiltration of both tumor-associated T cells and activated dendritic cells [73]. It was, therefore, suggested by the group that that DAMPs, like HMGB1, may activate dendritic cells which, in turn, elicit T cell proliferation and maturation and contribute to antitumor immune responses [73].

## Prospects for pyroptosis in anticancer therapy

**Fig. 2:**
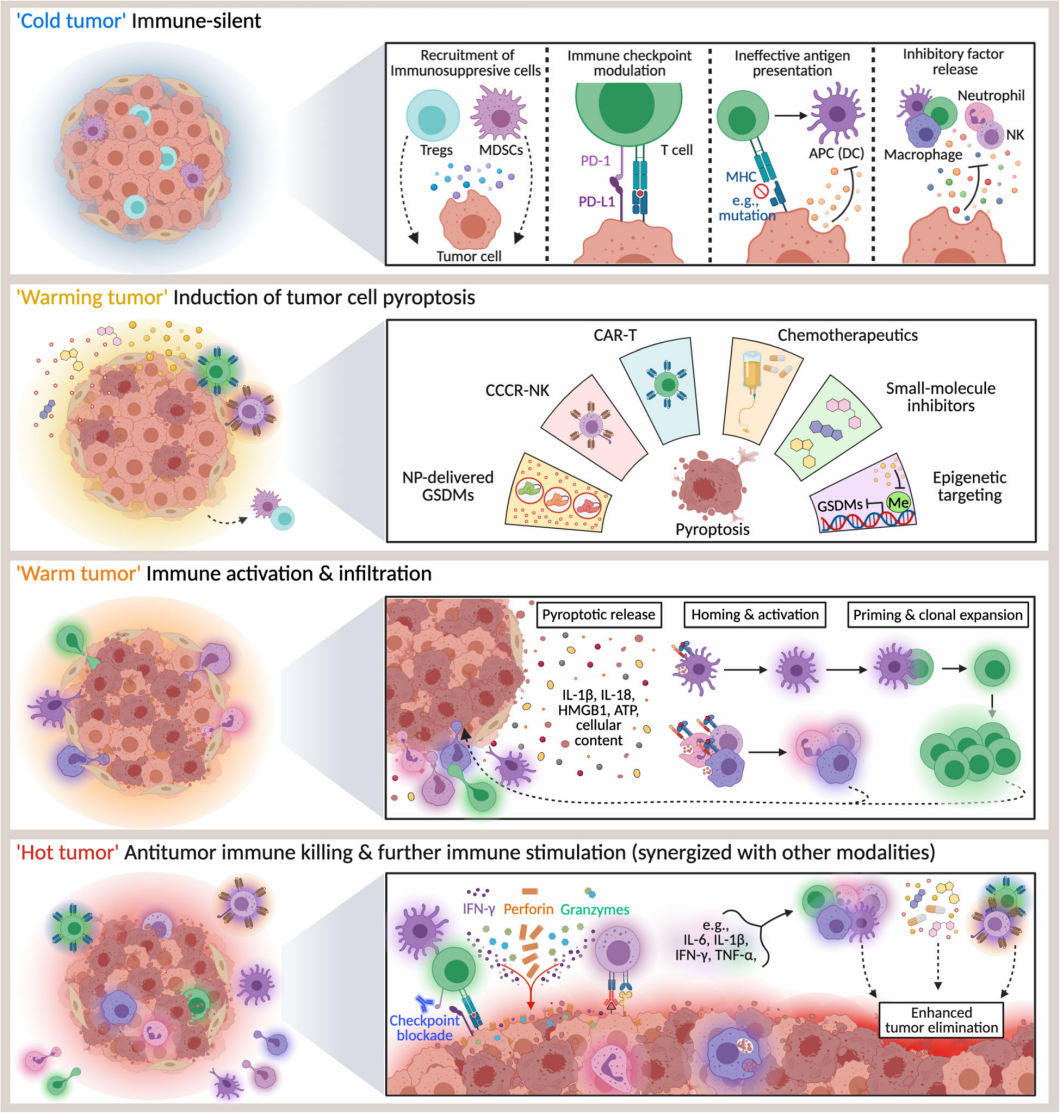
Pyroptosis heats anticancer immunity. ‘Cold tumor’: tumor cells create an immune tolerant microenvironment and avoid immune detection and killing by recruiting immunosuppressive cells, increasing immune checkpoint proteins, impeding antigen presentation, and releasing immune inhibitory factors. ‘Warming tumor’: various strategies are used to induce tumor cell pyroptosis and “heat” tumors from immune-silent states. ‘Warm tumor’: pyroptotic tumor cells release pro-inflammatory cytokines and immunogenic material that prompt immune cell activation and recruitment. ‘Hot tumor’: infiltrated immune cells recognize and kill tumor cells, and this killing may participate in a positive feedback loop that enhances tumor-specific immunity. Tumor elimination may be further increased through combinatorial therapeutic strategies. CAR-T, chimeric antigen receptor T cell; CCCR-NK, chimeric costimulatory converting receptor natural killer cell; DC, dendritic cell; GSDMs, gasdermin proteins; HMGB1, high-mobility group box protein 1; IFN-γ, interferon-gamma; IL, interleukin; MDSCs, myeloid-derived suppressor cells; MHC, major histocompatibility complex; NK, natural killer cell; NP, nanoparticle; PD-L1, programmed death-ligand 1; PD-1, programmed cell death protein 1; TNF-α, tumor necrosis factor-alpha; Tregs, regulatory T cells

In recent years, a growing number of studies have illustrated the feasibility and therapeutic potential of harnessing pyroptosis to engage antitumor immunity through diverse targeting and delivery methods (Fig. 2). Using tumor-cell-derived microparticles (TMP), for example, Gao et al. have delivered methotrexate into cholangiocarcinoma (CCA) cells to induce GSDME-mediated pyroptosis, leading to the activation of patient-derived macrophages and the recruitment of neutrophils to the tumor site for drug directed tumor destruction [74]. Moreover, when this methotrexate-TMP delivery system was infused into the bile duct lumen of extrahepatic CCA patients, neutrophil activation and resolution of biliary obstruction were observed in 25% of the patients [74]. GSDME-mediated pyroptosis has also been found to be prompted in melanoma through a combination of BRAF and MEK inhibitors, causing immune cell infiltration/activation and melanoma regression [73]. In another strategy, metformin, the most common drug used to treat type 2 diabetes, was used to inhibit cancer cell proliferation by indirectly activating pyroptosis through caspase-3 [75]. Specifically, metformin contributed to mitochondrial dysfunction and activated the AMPK/SIRT1/NF-κB pathway, promoting Bax accumulation and cytochrome c release, which, in turn, led to caspase-3 activation and GSDME cleavage [75].

A range of small-molecule inhibitors targeting KRAS-, EGFR-, or ALK-mutant lung cancers were also discovered to induce pyroptotic death through caspase-3-mediated cleavage of GSDME following activation of the mitochondrial intrinsic apoptosis pathway [43]. The group’s finding suggested that these two PCD pathways regulate each other and that pyroptosis may be used to increase the efficacy of anticancer targeted therapies, although this effect is reduced when apoptotic function is intact [43]. In breast cancer cells, treatment with a RIG-1 agonist triggered the extrinsic apoptosis pathway and pyroptosis, activating STAT1 and NF-κB and upregulating lymphocyte-recruiting chemokines [76]. Accordingly, a decrease in breast cancer metastasis and tumor growth was accompanied by an increase in tumor lymphocytes following RIG-1 activation in mice [76]. Although the switch from apoptosis to pyroptosis has yet to be fully elucidated, a recently synthesized NF-κB inhibitor, 13d, was found to arrest cancer cells in the G2/M phase and to promote this switch [77]. Treatment with 13d also produced a robust antitumor effect in vivo while exhibiting low toxicity [77], similar to L61H10, another compound reported to induce an apoptosis-to-pyroptosis switch, also likely through NF-κB inhibition [78].

One notable hurdle in developing pyroptosis-based anticancer strategies is the fact that many cancers significantly downregulate their expression of GSDM proteins or express mutated, non-functional forms of them [33]. Fortunately, this dilemma has captured the interest of many researchers who have begun to develop clever solutions, like Fan et al., who approached the problem through epigenetic targeting [79]. By using decitabine to demethylate *GSDME* in combination with nanoliposomes carrying chemotherapy drugs that activate caspase-3, the group effectively reversed *GSDME* silencing in tumor cells and induced pyroptosis. In addition to suppressing tumor growth, metastasis, and recurrence, this regimen also stimulated immune responses through pyroptosis-induced cytokine release [79]. Considering that 91% of the cancer patient-related *GSDME* mutations evaluated by Zhang et al. were seen to cause loss of function [33], however, it is suggestive that epigenetic targeting may not be an effective method to induce pyroptosis in certain patients. The targeted delivery of functional GSDM proteins directly to cancer cells via nanotechnology [70], may provide a reliable and effective way to circumvent this dilemma though.

Another major obstacle facing nearly all anticancer immunotherapeutic strategies is the dysregulation stemming from the immunosuppressive tumor microenvironment, such as through inhibitory receptors like PD-1. To address this, Lu et al. engineered NK92 cells containing a chimeric costimulatory converting receptor (CCCR) that converts the inhibitory PD-1 signal to an activating signal, effectively enhancing the cells’ antitumor activity against H1299 lung cancer cells [80]. In vitro, CCCR-NK92 cells rapidly killed H1299 cells through GSDME-mediated pyroptosis and, in vivo, significantly inhibited tumor growth [80]. Taken alongside Liu and colleagues’ observations of CAR-T cell-induced pyroptosis [72], it appears that future exploration into CAR-based therapies, though challenging, will be especially worthwhile. Moreover, the exciting and growing number of reports that pyroptosis induction synergizes with PD-1 inhibitors to turn ‘cold’ tumors ‘hot’ suggest that we have only begun to understand the combinatorial potential of pyroptosis (Fig. 2) [35, 70].

## Conclusions and future perspectives

As an inflammatory cell death mode, pyroptosis plays an important role in tumor suppression by galvanizing antitumor immune responses into action. In some instances, it is suggestive that pyroptosis induction alone may be sufficient to hinder tumor growth, although variability in its effectiveness and associated adverse effects (e.g., CRS in CAR-T cell therapy) hints that its clinical employment will likely be most effective when used in combination with other anticancer modalities and tailored to individual patients and cancers. One of the greatest challenges facing the therapeutic employment of pyroptosis appears to be the irregularity in expression and function of pyroptosis-related components, not only across different cancers but within them. Nonetheless, advances in molecular, genetic, and epigenetic targeting/delivery systems, alongside precision and personalized medicine, provide hope that we may soon possess the tools and knowledge needed to harness these powerful mechanisms as weapons against cancer.

## Data Availability

Not applicable.

## Abbreviations

AIM2: absent in melanoma 2
ASC: apoptosis-associated speck-like protein containing CARD
CAR: chimeric antigen receptor
CCA: cholangiocarcinoma
CCCR: chimeric costimulatory converting receptor
CRC: colorectal cancer
CRS: cytokine release syndrome
CTLs: cytotoxic T lymphocytes
DAMPs: damage-associated molecular patterns
eGFP: enhanced green fluorescent protein
GC: gastric cancer
GSDM: gasdermin
GSDMD-N: N-terminal domain of GSDMD
GzmA: granzyme A
GzmB: granzyme B
HCC: hepatocellular carcinoma
HMGB1: high-mobility group box protein 1
ICD: immunogenic cell death
IFN-γ: interferon-gamma
IL: interleukin
LPS: lipopolysaccharide
LUAD: lung adenocarcinoma
LUSC: lung squamous cell carcinoma
MLKL: mixed lineage kinase domain-like pseudokinase
NK: natural killer
NLRP1/3/4: NLR family pyrin domain-containing 1/3/4
NLRs: NOD-like receptors
NP: nanoparticle
NSCLC: non-small cell lung cancer
PAMPs: pathogen-associated molecular patterns
PCD: programmed cell death
PD-1: programmed cell death protein 1
PD-L1: programmed-death ligand 1
Phe-BF3: phenylalanine trifluoroborate
PRRs: pattern recognition receptors
TAMs: tumor-associated macrophages
TCGA: The Cancer Genome Atlas
TILs: tumor-infiltrating lymphocytes
TLRs: toll-like receptors
TMP: tumor-cell-derived microparticle
TNF-α: tumor necrosis factor-alpha
